# Malnutrition among 6–59-Month-Old Children at District 2 Hospital, Ho Chi Minh City, Vietnam: Prevalence and Associated Factors

**DOI:** 10.1155/2019/6921312

**Published:** 2019-02-05

**Authors:** Giao Huynh, Quynh H. Ngoc Huynh, Ngoc Han T. Nguyen, Quang Thanh Do, Van Khanh Tran

**Affiliations:** ^1^Faculty of Public Health, University of Medicine and Pharmacy at Ho Chi Minh City, Vietnam; ^2^Tien Giang General Hospital, Member of Vietnam Young Physician Association (VYPA), Vietnam; ^3^Dist 2 Hospital, Member of Vietnam Young Physician Association (VYPA), Vietnam

## Abstract

**Objectives:**

Childhood malnutrition is major health concern in many low- and middle-income countries, including Vietnam. It was a major risk factor for child mortality and adult ill-health. Malnutrition could increase the risk of serious infections; conversely current diseases also had a negative impact on the growth of child. This study, therefore, examines the prevalence of stunting and underweight among 6–59-month-old outpatient children in District 2 Hospital, Ho Chi Minh City, Vietnam.

**Methods:**

A cross-sectional study involved a sample of 225 children aged 6–59 months who were randomly selected from the Outpatient Department in District 2 Hospital. Anthropometric measurements and blood test of children were taken to assess the nutritional status and anaemia. A structured questionnaire was also used to collect mothers' and children's characteristics to examine associated risk factors.

**Results:**

The prevalence of stunting, underweight, overweight, and anaemia among children aged 6–59 months was 9.8%, 8.4%, 25.8%, and 30.7%, respectively. Underweight significantly correlated only to having breastfeeding in the first hour after birth (RR: 0.02; 95% CI: 0.01-0.17; p<0.001), while stunting was related to age of starting complementary foods from equal to/more than 6 months (RR=0.70, 95%CI=0.50-0.99, p<0.05) and normal birth weight (RR = 0.29, 95%CI = 0.15-0.56, p<0.001).

**Conclusions:**

This study emphasized the importance of measuring the overall nutritional status for children, who have coexisting infectious diseases and anaemia. The high prevalence of malnutrition and anaemia underlined the need for routine screening as well as treatment of children. Additionally, health information strategies should be focused on young children feeding practices to minimize stunting and underweight.

## 1. Introduction

Malnutrition was one of the most significant child health issues in developing countries. In 2012, approximately 19.4% and 29.9% of children aged under 5 years had underweight and stunting, respectively [1], with more than 3.4 million mortality cases among children aged under 5 years related to nutritional status [2]. World Health Organization (WHO) reported that the prevalence of stunting and underweight among children aged under 5 years globally in 2017 decreased; those were 13.5% and 22.2%, respectively. However, more than half of all stunted children under 5 years lived in Asia and Africa [3, 4]. Stunting refers to a child who is too short for his or her age; these children could suffer severe irreversible physical, cognitive damage and these devastating effects can last a lifetime and even affect the next generation [5]. Malnutrition was also one of the important risk factors in the onset of many communicable and noncommunicable diseases in both children and adults worldwide [6]. Therefore, adequate nutrition during infancy and early childhood is essential to ensure the growth, health, and development of children to their full potential [7]. Malnourished children suffer in higher proportion from respiratory infections, diarrhea, and measles, characterized by a protracted course and exacerbated disease [8]. Stimulation of an immune response by infection increased the demand for metabolically derived anabolic energy and associated substrates, leading to a synergistic vicious cycle of adverse nutritional status and increased susceptibility to infection [9].

Many national surveys showed that Vietnam achieved the target to reduce the prevalence of underweight. However, underweight and stunting remained at the level of moderate to high public health impact in 2010 (17.5%, 29.3%, respectively) and in 2014 (12.0%, 23.3%, respectively) [10, 11], while the percentage of children hospitalized at national hospital of pediatrics in Vietnam having underweight was higher (18.2%) [12]. Population-based information was available on malnutrition in under-5-year-old children in some national surveys; however, data on malnutrition and associated factors at hospital settings were rare. Thus, this study aimed to describe the rates of malnutrition among children aged 6–59 months in the Outpatient Department of District 2 Hospital in Ho Chi Minh City.

## 2. Materials and Methods

### 2.1. Setting and Study Population

A cross-sectional survey was conducted during January to July 2018 at District 2 Hospital. District 2 Hospital is located in the North East of Ho Chi Minh City. The average number of patients who visit pediatricians at District 2 Hospital was 80 per day. Among them, 50 were children aged 0 to 59 months. Children from 6 to 59 months old were systematically randomly selected from the daily registered list of patients with k=5 until we got 225 children. Besides, children's mothers or caregivers were also invited to interview session.

### 2.2. Data Collection

Children length/height and weight were collected by using a pediatric digital scale and pediatric anthropometric rule. Besides, anthropometric data of these children at birth were also obtained with the children's card of birth. Moreover, a structural questionnaire was also applied to interview children's mothers or caregivers about associated risk factors. Blood samples were collected after interviews. Hemoglobin (Hb) concentration was measured using blood samples taken from children's venous blood; two mL of blood was drawn into ethylenediaminetetraacetic acid (EDTA) tubes and analyzed using CELL–DYN Ruby Hematology Analyzer from Abbott.

### 2.3. Ethical Approval

Informed consent was obtained from all participants. This study was approved by the Ho Chi Minh City University of Medicine and Pharmacy, Vietnam (protocol number 165/UMP-BOARD). Coding was applied to ensure the participant anonymous.

### 2.4. Data Analysis

Data on height and weight were converted to z-scores of height/length-for-age (HAZ), weight-for-age (WAZ), weight-for-height/length (WHZ), and child body mass index (BMI) using the WHO Anthro software 3.2.2 [13]. The WHO standard reference was adopted to classify children nutritional status such as stunting (HAZ < -2) and underweight (WAZ < -2), and those with BMI-z-score > +1 were considered as at risk of overweight [14]. Hemoglobin (Hb) concentrations were performed using CELL–DYN Ruby Hematology Analyzer at District 2 Hospital, and anaemia was defined as Hb concentrations less than 110 g/L [15, 16].

Data which were collected by questionnaire were entered into EpiData 3.0 by two experienced research assistants. For ensuring data quality, double entry and 10% random check were applied. Stata for Window version 13 was used to analyze data. Descriptive statistics with frequency, percentage, mean scores, and standard deviation were performed. The Chi-square test and t-test were used to determine the relationship between factors and undernutrition status. Multivariate analysis was performed using Poisson regression for variables that had a significance level < 0.20 in the bivariate analysis.

## 3. Results

### 3.1. Characteristics of Participants

Table 1 showed the characteristics of 225 participants, including mothers and children aged 6.0–59.0 months. Nearly two-thirds of mothers were 25.0–<35.0 years old. Half of them were sellers (58.3%). Few of them had high school degree (5.8%). Most of them had moderate income (91.6%). In terms of children, most of children (93.3%) had normal birth weight. One-third of children were 36.0–59.0 months old with not much difference between genders. The majority of children (88.4%) had breastfeeding in the first hour after birth, but lower exclusive breastfeeding for the first 6 months of life (34.2%). 81.3% of children were recorded with acute respiratory infections. Besides, 9.8% and 30.6% of children came to hospital because of diarrhea and anaemic, respectively.

### 3.2. Prevalence of Underweight, Stunting, and Associated Risk Factors

Table 2 showed the malnourished children 9.8 % were stunted (low length/height-for-age), while 8.4% were underweight (low weight-for-age), and 25.8 % were at risk of overweight and overweight/obesity.

Characteristics of children with and without underweight were similar. However, having breastfeeding in the first hour after birth, getting full vaccination, and having diarrhea, as well as the duration of this disease, all correlated with significantly different underweight rate (p<0.05) (Table 3). Children who had breastfeeding in the first hour after birth had lower underweight rate than children without breastfeeding in the first hour (5.1% vs. 34.6%, p<0.01). Correspondingly, children who were fully vaccinated in EPI had lower underweight rate than those not getting full vaccination (6.5% vs. 25.0%, p<0.05). The rate of children diagnosed with diarrhea was higher than the rate of those who were not (22.7% vs. 6.9%, p<0.05), and the average number of days of this disease (3 days) was significantly correlated with underweight status (p<0.05). This study included 225 mothers; most mothers' characteristics did not have a statistically significant correlation with underweight rate of infants (p>0.05). The results of Poisson regression of factors associated with underweight status were summarized in Table 4. A statistically significant relationship between underweight rate and characteristics of infants, such as breastfeeding in the first hour after birth, was found. When model was adjusted for all other variables, there was a 0.01–0.17-fold decrease in underweight rate of children for a 1-unit increase in “breastfeeding in the first hour after birth” (p<0.01).

Table 5 indicated that characteristics of children and mothers with and without stunting were similar. However, having low birth weight (<2500g), breastfeeding in the first hour after birth, getting full vaccination, having diarrhea disease, and the existence of three or more children in household all correlated with significantly different stunting rate (p<0.05). Children who had low birth weight had higher stunting rate than children with normal weight at birth (33.3% vs. 8.1%, p<0.05). Besides, children breastfeeding in the first hour after birth had lower stunting rate than children without breastfeeding in the first hour (6.5% vs. 34.6%, p<0.001). Children getting full vaccination had lower stunting rate compared with those not getting full vaccination (6.9% vs. 33.3%, p<0.001). Additionally, the rate of children diagnosed with diarrhea was higher than the rate of those who were not (27.3% vs. 7.9%, p<0.05). Characteristics of mothers, including number of children in household, had a significant correlation with stunting (p<0.05).

Summarizing the results of Poisson regression model (Table 6), we found statistically significant relationship between characteristics of children such as birth weight and age of starting complementary foods with stunting rate. When the model was adjusted for all other variables, there was a 0.15–0.56-fold decrease in stunting rate of children for a 1-unit increase in “normal birth weight” (RR=0.29, 95%CI=0.15-0.56, p<0.001) and a 0.50–0.99-fold decrease in stunting rate of children for a 1-unit increase in age of starting complementary foods equal to/more than 6 months (RR=0.70, 95%CI=0.50-0.99, p<0.05).

## 4. Discussion

The results of this study suggested that malnutrition remains a problem in outpatient children, who had existing diseases and anaemia. They showed the double burden of malnutrition and disease. This “double burden” occurred in the same individual at different stages of his or her life, with the rates of 8.4% underweight, 9.8% stunting, 4.4% wasting, and 25.8% overweight. Proportions of stunting and underweight in our study were higher than those at Bagamoyo District Hospital in Tanzania, being 8.4% and 5.7%, respectively [17], and among hospitalized children in the Netherlands with 9% chronic malnutrition [18], but lower than that among hospitalized children in Romania with 13.6% stunting [19], as well as children hospitalized at National Hospital of Pediatrics in Vietnam having 18.2% underweight and 22.5% stunting [12]. There could be another subject and outpatient with milder level and shorter duration of current disease (3.2 ± 2.1 days). Therefore, all of these factors affected lower nutritional status than hospitalized children in another study. However, the proportions in our findings were higher than those in healthy children who had the same age (6–59 months) in Ho Chi Minh City in 2015 (7.0% stunting and 4.9% underweight) [20]. Because malnutrition led to a synergistic vicious cycle of adverse nutritional status and increased susceptibility to infection, there was currently different gap between malnutrition proportion in hospital and community populations under five. Our result showed high anaemia rate (30.6%). Prior studies conducted in Vietnam seemed to focus only on assessing frequency of anaemia in the large community; for example, Nguyen PH and Nhien NV's researches showed the prevalence of anaemia was high among children aged 0 to 59 months in Vietnam in 2006 (45.1%) [21] and 45% among primary school children in rural Vietnam [22]. We were unable to find any studies that evaluate malnutrition accompanied with anaemia in outpatient children. Therefore, we did not have any database for comparison. Because anaemia and malnutrition often had common causes, they indicated that malnutrition, anaemia problems, and infectious diseases would cooccur in the same individuals. Actually, our finding showed that children with anaemia had also higher stunting than those without (15.8% vs. 9.3%) and underweight was similar (13.2% vs. 3.5%). However, the comparison of observed and expected values was not significantly difference, maybe due to loss of power when we categorized Hb, HAZ, and WAZ as dichotomous variables. It was recommended that pediatricians needed a comprehensive screening of nutritional and anaemic status along with current diseases to intervene at the same time. Our result found that children aged 6-12 months had the highest stunting rate (17.1%) (Table 5). However, the comparison of observed and expected values did not show significant difference. De Novaes Oliveira M and Rannan-Eliya RP's researches on Brazilian children attending daycare centers [23] and Sri Lanka [24] had similar results. However, this was inconsistent with others, such as a study in rural Ethiopia showing that the proportion of child stunting increased as the age of the child increased [25]. There was statistically significant relationship between underweight rate and breastfeeding in the first hour after birth; that is, a decrease in underweight rate of children correlated to an increase in “breastfeeding in the first hour after birth” (Table 4), which was consistent with WHO's recommendation that breastfeeding should be done as soon as possible. Therefore, nutritional counseling to mothers was designed to promote breastfeeding practices. We also found that stunting was associated with a lower birth weight and age of starting complementary foods timely (equal to/more 6 months); that is, a decrease in stunting rate of children correlated to an increase in “normal birth weight”, with low birth weight negatively impacting stunting, and a decrease in stunting rate of children correlated with an increase in age of starting complementary foods timely. Therefore, education programs for mothers had to encourage starting complementary feeding along with breastfeeding for infants aged 6 months or more, and breastfeeding as soon as possible in the first hour after birth. Our study found that there was no association between characteristics of mothers and malnutrition status of children aged 6–59 months. This result was inconsistent with finding of Gwatkin and colleagues, which was high level of malnutrition among children of less educated women in developing countries [26]; in addition, Reed reported that higher level of education is associated with improved child weight-for-age [27].

### 4.1. Limitation

This study had limitations that should be considered in interpreting the results. It was difficult to generalize our hospital-based study's results to the general population. The limitation of a cross-sectional study in teasing out the cause-effect relationship between demographic characteristics of mothers, children, and malnutrition was considered. There was also possibility of social desirability bias; however, interviewers encouraged parents to express their opinions freely. In addition, though being essential to diagnose anaemia, hemoglobin measurement alone could not determine the cause of the anaemia. Future studies could conduct additional measurements of iron status such as serum ferritin or serum transferrin receptor.

## 5. Conclusion

This study emphasized the importance of measuring the overall nutritional status for children, who have coexisting infectious diseases and anaemia. The high malnutrition and anaemia prevalence underlined the need for routine screening as well as treatment in outpatient children. Additionally, health information strategies should be focused on young children feeding practices to minimize stunting and underweight.

## Figures and Tables

**Table 1:** Characteristics of participants (n=225).

**Characteristics of mothers**	N (%)
Mother's age (years)	
≥ 18 and <25	35 (15.6)
≥ 25 and <35	151 (67.1)
≥ 35	39 (17.3)
Occupation	
Housewife	39 (17.3)
Government officer/office worker	55 (24.4)
Seller	131 (58.3)
Education	
Primary school	101 (44.9)
Secondary school	111 (49.3)
High school and higher	13 (5.8)
Gross household income	
Moderate	206 (91.6)
Poor, near-poor households	19 (8.4)
Number of children in household	
1	87 (38.7)
2	111 (49.3)
≥ 3	27 (12.0)

**Characteristics of children**	N (%)

Gender (Male)	116 (51.6%)
Age (months)	
6 - 11	41 (18.2)
12 - 23	62 (27.6)
24 - 35	47 (20.9)
36 - 59	75 (33.3)
Nutrition history	
Breastfeeding in the first hour after birth (yes)	199 (88.4)
Exclusive breastfeeding for the first 6 months of	77 (34.2)
life (yes)	
Age of starting complementary foods (≥6 months)	14 (6.4)
*(n=219)*	
Getting full vaccination (yes)	201 (89.3)
Anaemia*(n=124)*	38 (30.6)
Existing diseases	
Acute respiratory infections	183 (81.3)
Diarrhea	22 (9.8)
Normal birth weight (≥ 2500 g)	210 (93.3)

**Table 2:** Prevalence of underweight and stunting (n=225).

**Nutrition status of children**	N (%)
Underweight (Weight-for-age: WA< -2SD)	19 (8.4)
Stunting (Length/height-for-age: HA< -2SD)	22 (9.8)
Wasting (Weight-for-length/height: WH< -2SD)	10 (4.4)
Overweight (BMI>+1)	58 (25.8)

**Table 3:** The association between underweight status and participants' characteristics (n=225).

	**Underweight**		p-value*∗*
	Yes (n=19)	No (n=206)	
**Characteristics of children**			
Gender			
Female	8(7.3)	101(92.7)	
Male	11(9.5)	105(90.6)	0.563
Age (months)			
6 - 11	4(9.8)	37(90.2)	
12 - 23	5(8.1)	57(91.9)	0.873
24 - 35	5(10.6)	42(89.4)	
36 - 59	5(6.7)	70(93.3)	
Weight at birth			
Low (< 2500g)	3(20.0)	12(80.0)	
Normal (≥ 2500 g)	16(7.6)	194(92.4)	0.121*∗∗*
Breastfeeding in the first hour after birth			
Yes	10(5.1)	189(94.9)	**<0.001**
No	9(34.6)	17(65.4)	
Exclusive breastfeeding for the first 6 months			
Yes	5(6.5)	72(93.5)	0.448
No	14(9.5)	134(90.5)	
Age of starting complementary foods *(n=219)*			
< 6 months	17(8.3)	188(91.7)	1.00*∗∗*
≥6 months	1(7.1)	13(92.7)	
Getting full vaccination			
Yes	13(6.5)	188(93.5)	**<0.05** *∗∗*
No	6(25.0)	18(75.0)	
Anemia (n=124)* missing 101*			
Yes	5(13.2)	33(86.8)	0.057*∗∗*
No	3(3.5)	83(96.5)	
Acute respiratory infections			
Yes	15(8.2)	168(91.8)	0.761*∗∗*
No	4(9.5)	38(90.5)	
Diarrhea			
Yes	5(22.7)	17(77.3)	**<0.05**
No	14(6.9)	189(93.1)	
Duration of this disease (days)	3(2-7)	2(2-3)	**<0.05** *∗∗∗*

**Characteristics of mothers**			
Age (years)			
≥ 18 and <25	4(11.4)	31(88.6)	
≥ 25 and <35	14(9.3)	137(90.7)	0.320
≥ 35	1(2.6)	38(97.4)	
Occupation			
Housewife	4(10.3)	35(89.7)	0.601
Government officer/office worker	6(10.9)	49(89.1)	
Seller	9(6.9)	122(93.1)	
Education			
Primary school	7(6.9)	94(93.1)	0.188
Secondary school	9(8.1)	102(91.9)	
High school and higher	3(23.1)	10(76.9)	
Gross household income			
Poor, near-poor households	2(10.5)	17(89.5)	
Moderate	17(8.3)	189(91.7)	0.067*∗∗*
Number of children in household			
1	11(12.6)	76(87.4)	
2	6(5.4)	105(94.6)	0.188
≥ 3	2(7.4)	25(92.6)	

Chi-square and *∗∗* Fisher exact tests used for comparison with and without underweight groups.

**Table 4:** Results of Poisson regression associated factors with underweight status.

**Variables**	**Risk Ratio**	**95**% **CI**	**p-value**
Breastfeeding in the first hour after birth	0.02	0.01-0.17	**<0.001**
Getting full vaccination	1.34	0.57 - 3.17	0.495
Diarrhea	0.18	0.01-5.08	0.314
Anemia	1.77	0.36 – 8.77	0.487
Duration of this disease	1.04	0.91 - 1.18	0.565

**Table 5:** The association between stunting status and participants' characteristics (n=225).

	**Stunting**		p value*∗*
	Yes (n=22)	No (n=203)	
**Characteristics of children**			
Gender			
Female	12(11.1)	97(88.9)	
Male	10(8.6)	106(91.4)	0.547
Age (months)			
6 - 11	7(17.1)	34(82.9)	
12 - 23	7(11.3)	55(88.7)	0.254*∗∗*
24 - 35	3(6.4)	44(93.6)	
36 - 59	5(6.7)	70(93.3)	
Weight at birth			
Low (< 2500g)	5(33.3)	10(66.7)	
Normal (≥ 2500 g)	17(8.1)	193(91.9)	**0.009**
Breastfeeding in the first hour after birth			
Yes	13(6.5)	186(93.5)	**<0.001**
No	9(34.6)	17(65.4)	
Exclusive breastfeeding for the first 6 months			
Yes	5(6.5)	72(93.5)	0.448
No	14(9.5)	134(90.5)	
Age of starting complementary foods *(n=219)*			
< 6 months	11(4.5)	65(85.5)	0.112
≥6 months	11(7.7)	132(92.3)	
Getting full vaccination			
Yes	8(33.3)	16(66.7)	
No	14(6.9)	187(93.1)	**<0.001**
Anemia (n=124)* missing 101*			
Yes	6(15.8)	32(84.2)	
No	8(9.3)	78(90.7)	0.293
Acute respiratory infections			
Yes	19(10.4)	164(89.6)	0.774*∗∗*
No	3(7.1)	39(92.9)	
Diarrhea			
Yes	6(27.3)	16(72.7)	**0,012**
No	16(7.9)	187(92.1)	
Duration of this disease (days)	3(2-4)	2(2-3)	0.089*∗∗∗*

**Characteristics of mothers**			
Age (years)			
≥ 18 and <25	5(14.3)	30(85.7)	
≥ 25 and <35	16(10.6)	135(89.4)	0.200
≥ 35	1(2.6)	38(97.4)	
Occupation			
Housewife	6(15.4)	33(84.6)	
Government officer/office worker	3(5.5)	52(94.6)	0.278
Seller	13(9.9)	118(90.1)	
Education			
Primary school	7(6.9)	94(93.1)	0.188
Secondary school	9(8.1)	102(91.9)	
High school and higher	3(23.1)	10(76.9)	
Gross household income			
Poor, near-poor households	3(15.8)	16(84.2)	0.357
Moderate	19(9.2)	187(90.8)	
Number of children in household			
1	15(17.2)	72(82.8)	
2	6(5.4)	105(94.6)	**<0.05** *∗∗*
≥ 3	1(3.7)	26(96.3)	

Chi-square, *∗∗* Fisher exact and t- tests used for comparison with and without stunting groups, excluding missing data.

**Table 6:** Results of Poisson regression of factors associated with stunting status.

Variables	**Risk Ratio**	**95**% **CI**	**p-value**
Normal birth weight	0.29	0.15-0.56	**<0.001**
Breastfeeding in the first hour after birth	0.78	0.29-2.07	0.612
Age of starting complementary foods timely	0.70	0.50-0.99	**<0.05**
Duration of this disease	1.12	0.90-1.39	0.323
Getting full vaccination	0.21	0.04-1.03	0.055
Diarrhea	0.87	0.10-7.52	0.902
Number of Children in household			
1	1		
2	0.36	0.14-1.02	0.055
3	0.12	0.16-1.70	0.054
Mother's age (years)			
<25	1		
≥ 25 and <35	1.39	0.68-2.83	0.363
≥ 35	0.46	0.06-3.48	0.451

## Data Availability

The primary data used to support the findings of this study are available from the corresponding author upon request.
